# A Bottom-Up Synthesis Approach to Silver Nanoparticles Induces Anti-Proliferative and Apoptotic Activities against MCF-7, MCF-7/TAMR-1 and MCF-10A Human Breast Cell Lines

**DOI:** 10.3390/molecules25184332

**Published:** 2020-09-22

**Authors:** Nurul Izzati Zulkifli, Musthahimah Muhamad, Nur Nadhirah Mohamad Zain, Wen-Nee Tan, Noorfatimah Yahaya, Yazmin Bustami, Azlan Abdul Aziz, Nik Nur Syazni Nik Mohamed Kamal

**Affiliations:** 1 Integrative Medicine Cluster, Advanced Medical and Dental Institute, Universiti Sains Malaysia, Bertam, Kepala Batas, Penang 13200, Malaysia; izzatizulkifli.niz@gmail.com (N.I.Z.); musthahimahmuhamad@yahoo.com (M.M.); nurnadhirah@usm.my (N.N.M.Z.); noorfatimah@usm.my (N.Y.); 2 Chemistry Section, School of Distance Education, Universiti Sains Malaysia, Gelugor, Penang 11800, Malaysia; tanwn@usm.my; 3 School of Biological Sciences, Universiti Sains Malaysia, Penang 11800, Malaysia; ybustami@usm.my; 4 School of Physics, Universiti Sains Malaysia, Penang 11800, Malaysia; lan@usm.my

**Keywords:** biosynthesis, silver nanoparticles, MCF-7, MCF-7/TAMR-1, MCF-10A, cytotoxicity, apoptosis

## Abstract

A bottom-up approach for synthesizing silver nanoparticles (AgNPs-GA) phytomediated by *Garcinia atroviridis* leaf extract is described. Under optimized conditions, the AgNPs-GA were synthesized at a concentration of 0.1 M silver salt and 10% (*w*/*v*) leaf extract, 1:4 mixing ratio of reactants, pH 3, temperature 32 °C and 72 h reaction time. The AgNPs-GA were characterized by various analytical techniques and their size was determined to be 5–30 nm. FTIR spectroscopy indicates the role of phenolic functional groups in the reduction of silver ions into AgNPs-GA and in supporting their subsequent stability. The UV-Visible spectrum showed an absorption peak at 450 nm which reflects the surface plasmon resonance (SPR) of AgNPs-GA and further supports the stability of these biosynthesized nanoparticles. SEM, TEM and XRD diffractogram analyses indicate that AgNPs-GA were spherical and face-centered-cubic in shape. This study also describes the efficacy of biosynthesized AgNPs-GA as anti-proliferative agent against human breast cancer cell lines, MCF-7 and MCF-7/TAMR-1. Our findings indicate that AgNPs-GA possess significant anti-proliferative effects against both the MCF-7 and MCF-7/TAMR-1 cell lines, with inhibitory concentration at 50% (IC_50_ values) of 2.0 and 34.0 µg/mL, respectively, after 72 h of treatment. An induction of apoptosis was evidenced by flow cytometry using Annexin V-FITC and propidium iodide staining. Therefore, AgNPs-GA exhibited its anti-proliferative activity via apoptosis on MCF-7 and MCF-7/TAMR-1 breast cancer cells in vitro. Taken together, the leaf extract from *Garcinia atroviridis* was found to be highly capable of producing AgNPs-GA with favourable physicochemical and biological properties.

## 1. Introduction

Nanoparticles are ultrafine particles with sizes of nanometer order, denoting the minus 9th power of ten, namely one billionth [1]. By this definition, particles whose sizes are between 1 to 100 nm are regarded as nanoparticles. The multifunctional effects of silver nanoparticles (AgNPs) have made these nanomaterials potent compounds for biomedical, agricultural and pharmaceutical applications [2,3]. AgNPs differ from bulk and micron size silver in their size, shape, and stability [4]. Several techniques are commonly employed to synthesize AgNPs, including chemical synthesis [5], electrochemical synthesis [6], radiation synthesis [7] and photochemical synthesis [8]. In comparison with other methods, biological synthesis of AgNPs has received a great interest due to its eco-friendly mode of synthesis with passable biomedical properties [9].

The ultrafine size of the nanoparticles manifests in useful functions. For example, finer particles are apt to be absorbed more easily through biological membranes [1]. The size and shape of AgNPs can be controlled through optimization of different parameters such as temperature, pH, precursors, reducing agents, and other experimental conditions [4]. It has been demonstrated that plant extracts, bacteria, fungi yeasts and algae can be used as reducing and/or stabilizing compounds to transform silver ions (Ag^+^) from silver salts into nanoparticles [10]. This approach has been actively pursued in recent years to address the drawbacks of other physiochemical methods [10]. Among the biological methods, the use of plant extracts for the synthesis of AgNPs have been preferred due to the fact it is a green, simple, rapid and economically scalable room temperature method [11]. Several plant extracts including *Ocimum sanctum* [12], *Rumex hymenosepolus* [13], *Alternanthera sessilis* [14], *Eleutherococcus senticosus* [15] and *Cassia auriculata* [16] have been used to produce “green” AgNPs.

*Garcinia atroviridis* (*G. atroviridis*) or locally known as “*asam gelugor*” among Malaysians, is used in a folk medicine for the treatment of abdominal pain, infections, gastric and pains associated with pregnancy [17,18]. The plant is native to the Peninsular Malaysia, Thailand, Myanmar and Indian regions [19]. The fruits, leaves, and stem bark extracts of *G. atroviridis* are found to be a rich source of bioactive secondary metabolites, including phenolic, flavonoid and tannin compounds such as xanthones (atroviridin), benzoquinones (atrovirinone), cambogin, garcinol, camboginal, triflavanone (garcineflavanone A) and biflavonols (garcineflavonol A) [20,21,22]. These phytochemicals have been reported to display diverse pharmacological activities including antioxidant [22,23], antimicrobial [23,24], anti-proliferative [17,23,25], anti-inflammatory [24], anti-cholinesterases [21] and anti-hyperlipidemic effects [20]. Interestingly, it has been reported that some of these phytometabolites such as phenolic compounds, were capable to transform inorganic silver ions into nanoparticles [10].

Breast cancer is one of the most common forms of cancer observed in women and the disease is commonly associated with prolonged exposure to estrogens [26]. The most acknowledged mechanism of estrogen carcinogenicity is through its binding to estrogen receptor α (ERα) [27]. This binding enhances production of growth factors through direct and/or indirect actions which led to breast cell proliferation [27]. Moreover, the percentage of ER-positive cells generally increases in proliferative benign disease which explains why about 50–80% of breast cancer cases are classified as ERα-positive [28]. Clinically, ERα-positive breast cancer is treated with antiestrogen therapy, such as tamoxifen, that is designed to interrupt the function of ERα [29]. On a molecular basis, tamoxifen binds to ERα and competitively inhibits estrogen binding in the breast [30]. However, antiestrogen resistance frequently occurs, that eventually leads to treatment failure, disease progression and death [30].

In the present study, silver nanoparticles (AgNPs-GA) was biosynthesized following a “bottom- up” approach [31] using *G. atroviridis* leaf water extract to react with silver salt. The main objectives of the present study were to biosynthesize silver nanoparticles using *G. atroviridis* and characterize their physicochemical properties, followed by an investigation of their anti-proliferative potential against human breast cancer cells in vitro. Further, induction of apoptosis was evaluated by comparing the effects of AgNPs-GA in MCF-7, MCF-7/TAMR-1 and MCF-10A cells in vitro.

## 2. Results and Discussion

### 2.1. Characterization of Biosynthesized Silver Nanoparticles

The following characterization methods were used to confirm the formation of AgNPs-GA biosynthesized using *G. atroviridis* leaf extract.

#### 2.1.1. Ultra Violet-Visible (UV-Vis) Spectroscopic Analysis of AgNPs-GA

In the present study, AgNPs-GA were biosynthesized using a previously reported method with a slight modification [32,33]. AgNPs-GA were thus biosynthesized through a series of optimizations of different reaction parameters including the concentrations of silver salt (AgNO_3_) and leaf extract, mixing ratio of reactants, temperature and pH. The optimized reaction conditions for the biosynthesis AgNPs-GA are summarized in Table 1.

**Figure 1:**
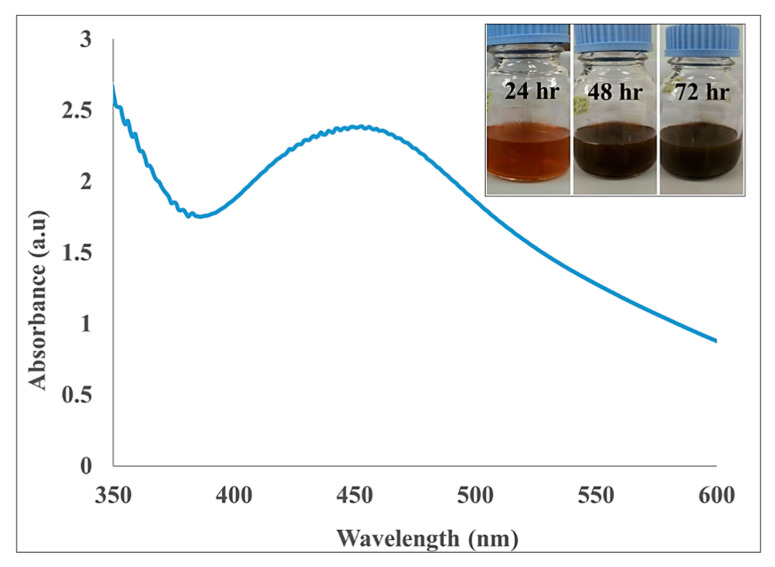
UV-Vis absorption spectrum recorded at optimum reaction condition and visible observation (insert) of biosynthesized AgNPs-GA at 24 h, 48 h and 72 h.

The optimal concentrations of leaf extract and aqueous AgNO_3_ were 10% (*w/v*) and 0.1 M, respectively and the best mixing ratio of both reactants was 1:4. In the present study, AgNPs-GA were formed after 72 h reaction time and at 32 °C. It is well-documented that AgNPs exhibit a yellowish brown color in aqueous solution due to excitation of surface plasmon vibrations in AgNPs, and the color started to change to dark brown due to reduction of silver ion, which indicated the formation of AgNPs [34]. In agreement with Ismail et al. [34], the color of AgNPs-GA samples was transformed to darker brown after 72 h of reaction time between the *G. atroviridis* leaf extract (Leaf-GA) and AgNO_3_ (Figure 1, insert).

AgNPs is known to possess optical properties which may interact with specific wavelengths of light [35]. The optical absorption spectra of AgNPs-GA were determined by UV-Vis spectrophotometry. The standard range of UV-Vis absorption maximum for AgNPs is described to be between 400 to 500 nm due to the excitation mode of their localized surface plasmon resonance (LSPR) [36]. The frequency and the strength of resonance are determined by the size and shape of the particles as well as the dielectric function of the surrounding medium [37]. The formation of AgNPs-GA occurred at pH 3.02, as displayed by a plasmon band at 450 nm, characteristic of nanosized silver, whilst broadening of peak indicated that the particles are polydispersed (Figure 1). This finding is in accordance with the reported literature [10]. It has been suggested that a larger number of functional groups that can bind and nucleate metal ions become accessible at pH 3.0 and 4.0, which resulted in small-sized metal nanoparticles [10].

#### 2.1.2. Scanning Electron Microscopy (SEM) Analysis

**Figure 2:**
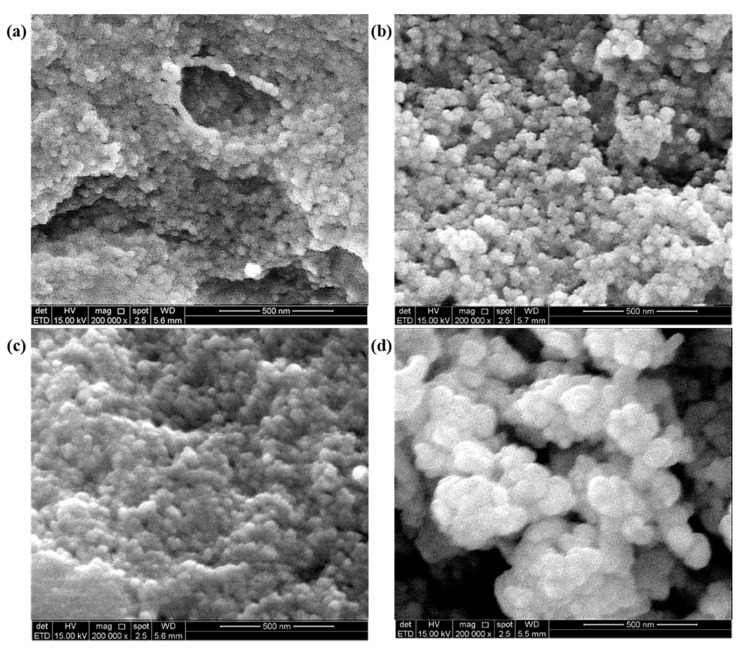
(**a**–**c**) SEM images of AgNPs-GA biosynthesis at 24 h, 48 h and 72 h of incubation period, respectively and (**d**) commercial AgNPs.

The morphology of the biosynthesized AgNPs-GA was analyzed using SEM. Representative SEM images of the control, commercial AgNPs and biosynthesized AgNPs-GA under optimum conditions at various incubation times (24, 48 and 72 h) are shown in Figure 2a–d.

Silver nanoparticles can be of different shapes such as spheres, rods, wires and triangles [38]. Based on our SEM analysis, spherical AgNPs were formed on the surface and most of the nanoparticles are aggregated as clusters as the incubation time increased from 24–72 h. This might due to cross-linking during sample preparation, thus resulting in the observed small variation of the nanoparticle size [39].

#### 2.1.3. Transmission Electron Microscopy (TEM) Analysis

**Figure 3:**
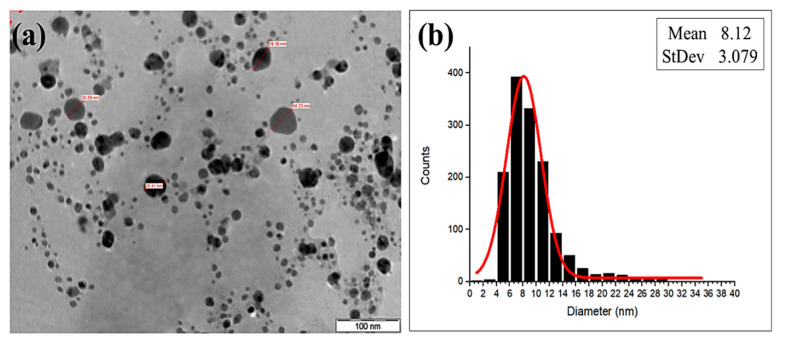
(**a**) TEM image of biosynthesized AgNPs-GA and (**b**) size distribution of biosynthesized AgNP-GA.

The size and shape of the biosynthesized AgNPs-GA under optimum conditions were examined using TEM analysis. The AgNPs-GA formed were predominantly spherical in shape (Figure 3a). Silver nanoparticles can be of different sizes, which range from a few up to 100 nm [38]. The particle size distribution histogram shows that the biosynthesized AgNPs-GA formed were in the range of 5–30 nm, with a mean value of 8.12 nm (Figure 3b). This finding indicated that the biosynthesis of AgNPs-GA results in a narrow distribution of well monodispersed nanoparticles. Previous reports had shown that the synthesis of plant mediated AgNPs are mostly spherical in shape [40,41]. It is interesting to note that a thin layer of organic materials from the plant is noticed on the TEM image. This could be attributed to the presence of biomolecules in the leaf extract, as reported by previous studies which employed the plant extracts in the biosynthesis [42]. This assumption was further validated by FTIR analysis.

#### 2.1.4. Dynamic Light Scattering (DLS) Analysis

**Figure 4:**
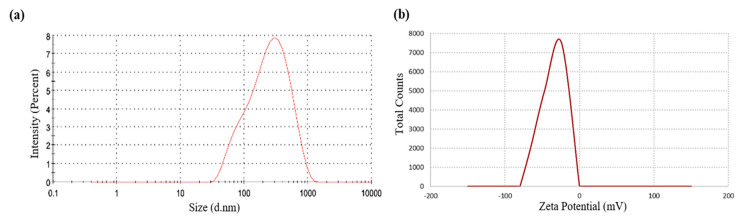
Dynamic light scattering analysis to determine size (**a**) and (**b**) zeta potential of AgNPs-GA.

The Z-average hydrodynamic diameters of AgNPs-GA was approximately 174.6 nm (Figure 4a) and zeta potential of the nanoparticles was −24.4 mV (Figure 4b) which can be defined as metastable. The polydispersity index (PDI) value of AgNPs-GA was found to be 0.4 that shows a moderate dispersity. A nanoparticle scheme with PDI value < 0.1 is measured as highly monodisperse, while PDI value > 0.4 and value in range of 0.1–0.4 are specified that the system has greatly polydisperse and moderately disperse distribution in the corresponding order [43]. Accordingly, biosynthesized AgNPs-GA display better particle size distribution characteristics.

The difference in the average crystallite diameter achieved from the Scherrer equation and Z-average hydrodynamic diameter from the DLS particle size analysis could be assigned that the hydrodynamic diameter is inclusive of any plant biomolecule coating on the nanoparticles, while the crystallite diameter is merely its core diameter [43]. In addition, the DLS-calculated size is slightly bigger as compared to the particle size calculated from TEM micrographs, which could be explained by the fact the DLS method result is calculated from the hydrodynamic radius [44].

In comparison to the observations obtained by TEM, the DLS analysis indicated that the size distribution of AgNPs-GA showed a broad distribution. This could be due to several factors such as hydrodynamic radius measurement and the intensity. Taken together, it could be suggested that AgNPs-GA is metastable and showed a good dispersity, in between monodispersed and polydispersed.

#### 2.1.5. X-ray Diffraction (XRD) Analysis

**Figure 5:**
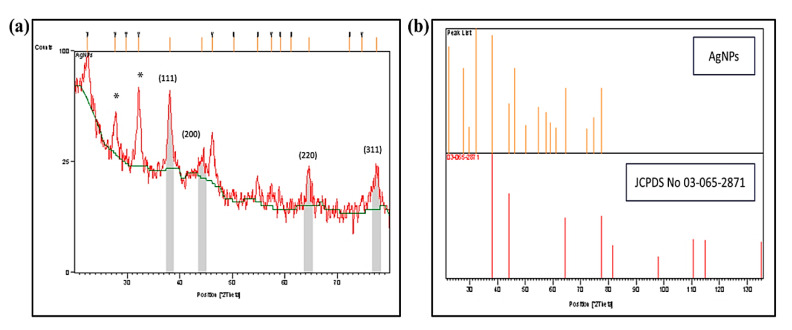
(**a**) XRD pattern of biosynthesized AgNPs-GA index at (111), (200), (220) and (311) and (**b**) a prominent peak between 30–130 degrees depicting the presence of silver in AgNPs-GA sample in comparison to XRD standard graph.

The crystalline nature of the biosynthesized AgNPs-GA was determined by XRD analysis in the range of 30–70° at 2θ angles (Figure 5a). The XRD spectrum showed four prominent diffraction peaks at 2θ values of 38.12°, 44.20°, 64.68°, and 77.55°, corresponding to (111), (200), (220), and (311) Bragg’s reflections planes of the faced-centered cubic (fcc) structure of metallic silver. The observed values are in good agreement with reference of fcc structure from Joint Committee of Powder Diffraction Standard (JCPDS No 03-065-2871) (Figure 5b), confirming the biosynthesis of AgNPs [45]. The average particle size of AgNPs-GA is determined using the Debye-Scherrer equation and the average particle size was found to be 14.64 nm. The additional peaks marked as (*) were also observed in the XRD spectrum. These peaks might due to the presence of the organic compounds in the leaf extract which contributed to the silver ions (Ag^+^) reduction and stabilization of the AgNPs (Ag^0^) formed [46].

#### 2.1.6. Fourier-Transform Infrared (FTIR) Analysis

**Figure 6:**
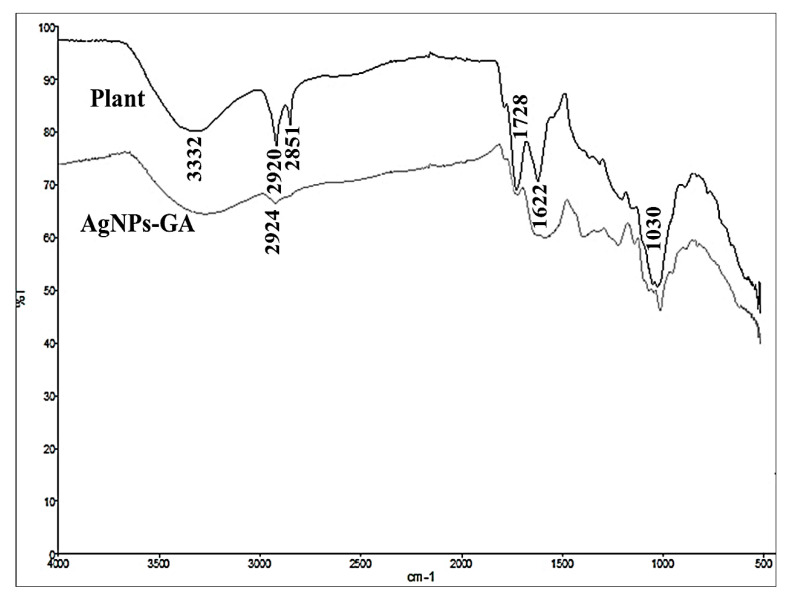
FTIR analysis of *G. atroviridis* leaf powder and biosynthesized AgNPs-GA.

Figure 6 shows the FTIR spectra of *G. atroviridis* leaf extract and its biosynthesized AgNPs-GA. In general, the FTIR spectrum of the extract exhibited major absorption bands at 3332 cm^−1^ (–OH stretching), 2920 and 2851 cm^−1^ (–CH stretching), 1728 cm^−1^ (–C=O stretching), 1622 cm^−1^ (–C=C– stretching) and 1030 cm^−1^ (–C–O stretching) [47,48]. The observed bands suggest the occurrence of flavonoids and phenolic compounds in the plant extract [21,48]. On the other hand, a majority of the absorption bands were present in the spectrum of AgNPs-GA with some marginal shifts, indicating the reduction of silver ions (Ag^+^) and the detection of some of the residual phenolic compounds from the extract [49]. Thus, the analysis suggested the dual role of *G. atroviridis* extract, which acts as the green reducing agent as well as the stabilizing agent in the formation of AgNPs [49].

### 2.2. Effect of AgNPs-GA on Cell Viability

**Figure 7:**
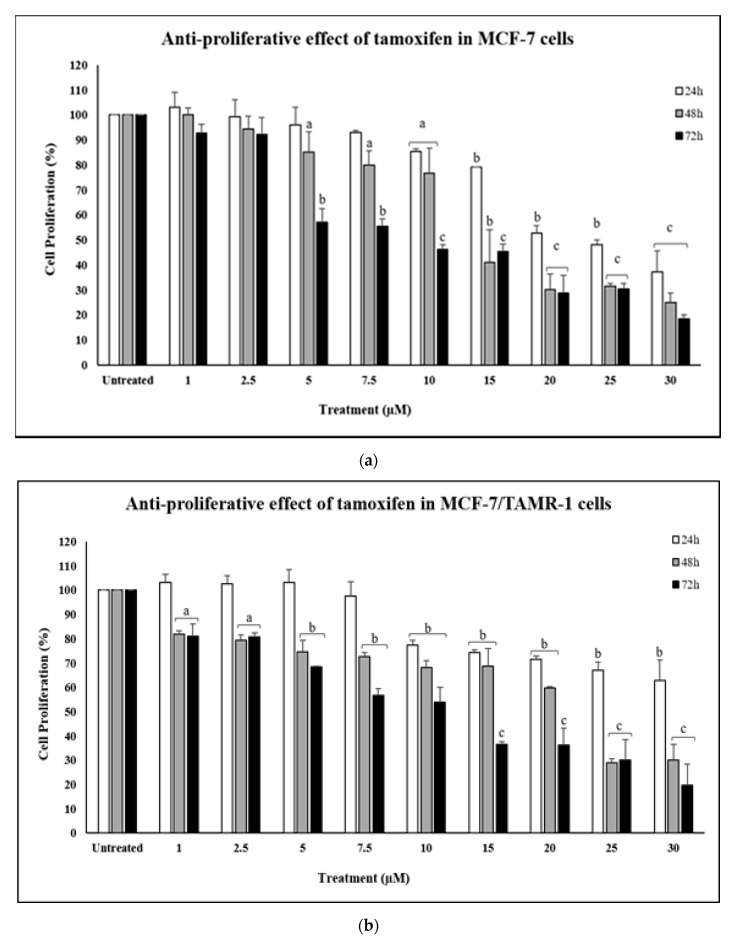

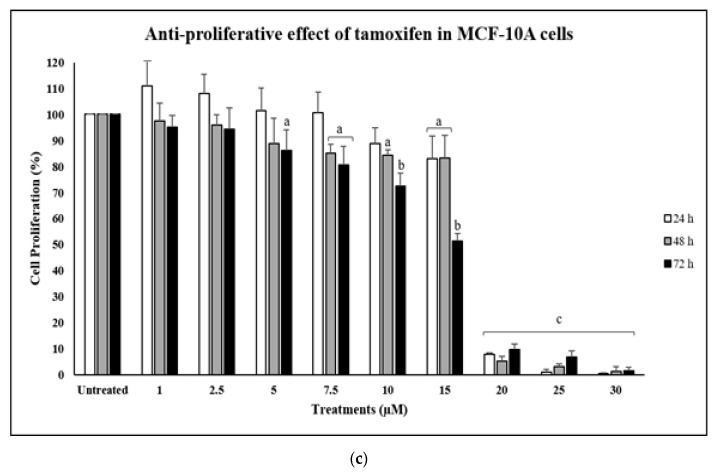
The anti-proliferative effect of tamoxifen on (**a**) MCF-7, (**b**) MCF-7/TAMR-1 and (**c**) MCF-10A cells within 24–72 h of treatment. The anti-proliferative effect of tamoxifen (1.0–30 µM) was assessed by MTT assay. Data shown are the mean values ± S.D. for three independent experiments. Statistical analysis was performed using Student’s *t* test with ^a^ *p* < 0.05, ^b^ *p* < 0.01 and ^c^ *p* < 0.001, significantly different to untreated cells.

The potential growth-inhibitory effect of biosynthesized AgNPs-GA were investigated against MCF-7 and MCF-7/TAMR-1 cell lines. Prior to the determination of the anti-proliferative effects of AgNPs-GA, both cell lines were treated with different concentrations of tamoxifen for up to 72 h and cell viability was measured by MTT assay in order to determine the degree of resistance acquired by MCF-7/TAMR-1 cells. When both cell lines were treated with 30 µM tamoxifen at 24 h, cell viability of MCF-7/TAMR-1 was significantly higher (62.7%) than that of MCF-7 (37.3%), demonstrating MCF-7/TAMR-1 cells are resistance to tamoxifen about 1.7 fold-different in comparison to MCF-7 cells (Figure 7a,b). MCF-10A cells were found to be more sensitive upon tamoxifen treatment, especially at concentrations 20–30 µM where the cell viability was reduced maximum (Figure 7c).

Tamoxifen is the first-line chemotherapeutic drug prescribed for ER-positive breast cancer patients. However, its use is hampered by the frequently occurring development of resistance during therapy and it has been the subject of intense study over recent years. Therefore, new strategies against these resistant cancer cells are urgently needed. In this study, we have primarily tested the efficacy of AgNPs-GA as potential anti-proliferative agent against ER-positive breast cancer cell lines, MCF-7 and MCF-7/TAMR-1.

Both cells were originally purchased from American Type Culture Collection (ATCC, Rockville, MD, USA) and Merck (Darmstadt, Germany), respectively. MCF-7/TAMR-1 is a tamoxifen-resistant cell line derived from the MCF-7/SO.5 cell line that has been adapted to grow at low serum concentration under the long-term treatment with 1 µM with tamoxifen. This cell line provides a model cell system for studying tamoxifen resistance, such as investigating and identifying new agent against tamoxifen-resistant growth. Tamoxifen resistant cells are characterized by less free sulfhydryl-groups (glutathione) [50]. Due to these insufficient amounts of glutathione, the authors had suggested that TamR cells were more sensitive to dicarbonyl stress. Dicarbonyl stress can result in damage to intracellular proteins, mitochondrial dysfunction and oxidative stress, which eventually leads to cell death [51,52].

Nass and colleagues [50] have also postulated that tamoxifen resistant cells are more sensitive to oxidative stress, which can be resulted by increasing endogenous production of ROS or a reduced expression of antioxidant defence systems [50]. Along with other studies, there is strong evidence for a link between AgNP-mediated production of ROS, the subsequent generation of oxidative stress and cytotoxicity [53,54]. For example, the biosynthesized AgNPs have been demonstrated to induce cytotoxicity in hepatocytes and fibroblasts due to oxidative damage to the cell membrane [54]. These findings prompted our interest to investigate whether AgNPs-GA can induce cell death in MCF-7/TAMR-1 cells as well.

**Figure 8:**
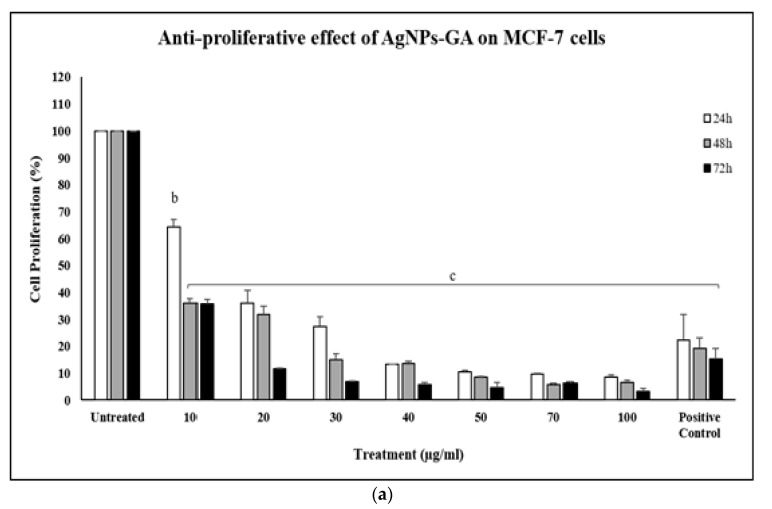

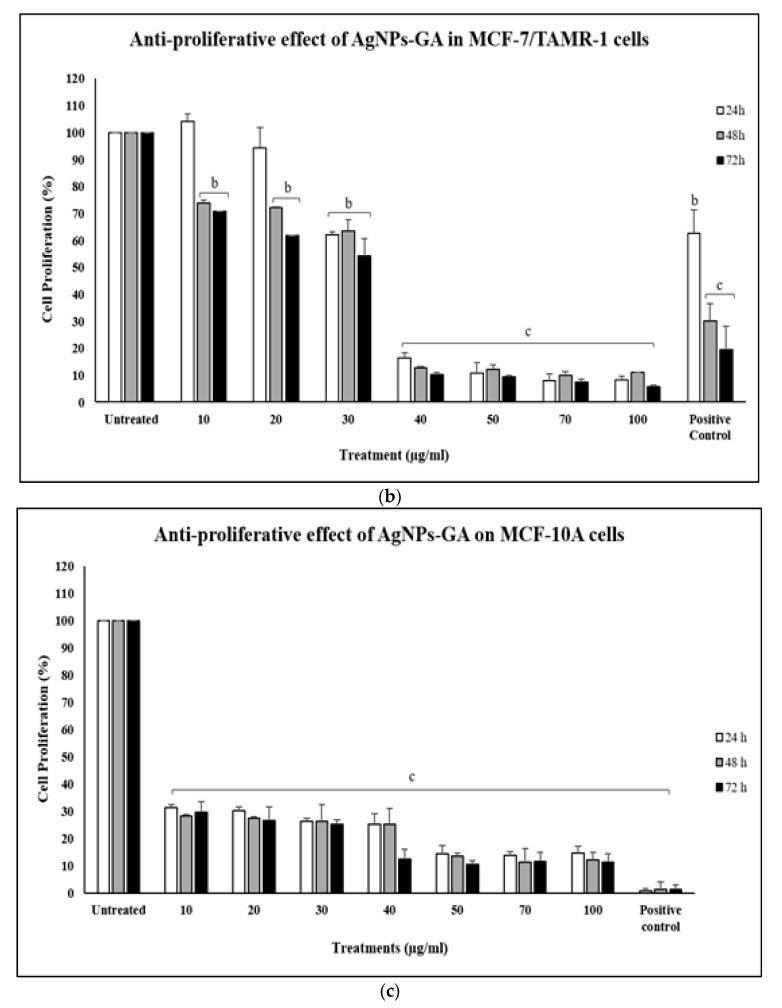
The anti-proliferative effect of biosynthesized AgNPs-GA on (**a**) MCF-7, (**b**) MCF-7/TAMR-1 and (**c**) MCF-10A cells within 24–72 h of treatment. The anti-proliferative effect of AgNPs-GA (10–100 µg/mL) was assessed by MTT assay. Tamoxifen (30 µM) was used as positive control. Data shown are the mean values ± S.D. for three independent experiments. Statistical analysis was performed using Student’s *t* test with ^a^ *p* < 0.05, ^b^ *p* < 0.01 and ^c^ *p* < 0.001, significantly different to untreated cells.

In this study, the anti-proliferative effects of AgNPs-GA at varying concentrations (10–100 µg/mL) were evaluated against MCF-7, MCF-7/TAMR-1 and MCF-10A human breast cell lines. The MCF-10A cell line was included as a representative of a human non-cancerous breast cell line. The obtained results at each incubation period (24–72 h) are illustrated in Figure 8a–c. Figure 8a shows clearly that increasing the concentration of AgNPs-GA from 10 to 100 µg/mL adversely decreased the proliferation of MCF-7 cells. At 24 h, MCF-7 cells remained viable about 65% and 10% after treated with 10 and 100 µg/mL of AgNPs-GA, respectively. The anti-proliferative effect of AgNPs-GA in MCF-7 cells also follows a time-dependent manner. After 72 h of incubation these cells were further inhibited by almost 97% by AgNPs-GA at concentration of 100 µg/mL. Compared with untreated cells, these differences in proliferation were statistically significant. Similar to MCF-7 cells, the anti-proliferative effect of AgNPs-GA against the MCF-7/TAMR-1 cell line also follows a concentration-dependent manner (Figure 8b). For instance, AgNPs-GA did not show significant anti-proliferative effects in MCF-7/TAMR-1 cells at concentrations lower than 20 µg/mL within a 24 h incubation period. A similar time-dependent response profile was also observed in MCF-7/TAMR-1 cells treated with AgNPs-GA. The viability of MCF-7/TAMR-1 cells was significantly decreased to 8.3%, 11.1% and 5.7% after treatment with a 100 µg/mL concentration of AgNPs-GA at 24, 48 and 72 h, respectively (Figure 7b). Compared with untreated cells, these differences in proliferation were statistically significant. The results also showed that AgNPs-GA reduced the viability of MCF-10A cells in a concentration-dependent manner (Figure 8c). As shown, AgNPs-GA displayed about 80% anti-proliferative activity at concentrations greater than 50 µg/mL. In vitro cell studies reported that the ability of AgNPs to cause toxicity and decrease membrane integrity is dependent on the type and size of cells [55]. In addition, the architectural differences between cancer and normal cells may also determine their specific elasticity and responses towards the potential toxicity possessed by AgNPs [56,57]. Geltmeier et al. had described that MCF-7 cells manifest higher elasticity and larger size compared to MCF-10A cells [56]. Furthermore, single cells of various cancer types were about two-fold softer than corresponding normal tissue cells [58]. Therefore, differences in the aforementioned cell modelling, like diameter, shape and volume of cells nuclei between MCF-7 and MCF-10 cells, at least in part, responsible for the differential cytotoxic effects of AgNPs-GA as observed in the present study. These findings suggest that MCF-10A cells are more susceptible to AgNPs-GA cytotoxicity, a potential vulnerability that could be improved in future studies. The most commonly described mechanism of cytotoxicity of AgNPs is through their silver ion release. Once a nanoparticle is located within a site, it has the ability to protractedly release silver ions, thus increasing the potential toxic impact [59]. For example, Kittler et al. reported that the release of silver ions led to a considerably increased toxicity of silver nanoparticles toward human mesenchymal stem cells [59]. Preclinical studies on biologically synthesized AgNPs and transactivator of transcription (TAT)-modified nanosilver possess cytotoxic activity toward MDA-MB-231 and MCF-7/ADR resistant cancer cells, respectively [60,61]. The physiochemical properties of AgNPs (e.g., size, shape, concentration) also play important roles to their cytotoxic effects. Generally, AgNPs are highly toxic at concentrations ranging from 5–10 µg/mL and sizes from 10–100 nm, which have been demonstrated by in vitro tests [4]. It can be assumed based on several studies which demonstrated nanosized particles are several times more catalytic, thus becoming more reactive [4].

For example, the high surface area-to-volume ratio enhances the surface properties of AgNPs, thereby increasing their interaction with cell membrane proteins and activate signalling pathways to generate reactive oxygen species (ROS) [4,62]. This ROS generation leads to damage of proteins and nucleic acids and finally causing apoptosis and inhibition of cell proliferation [4,62]. Furthermore, reactive oxygen species (ROS) generation capability could make them more toxic than their bulk counterparts [4,62].

**Figure 9:**
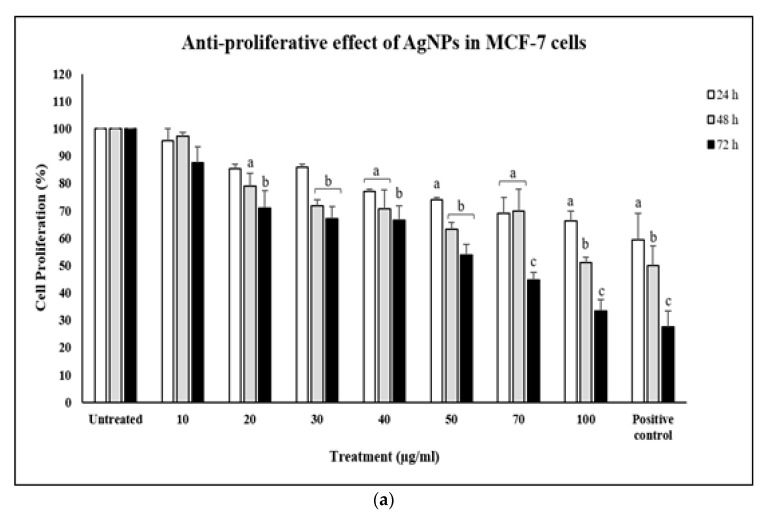

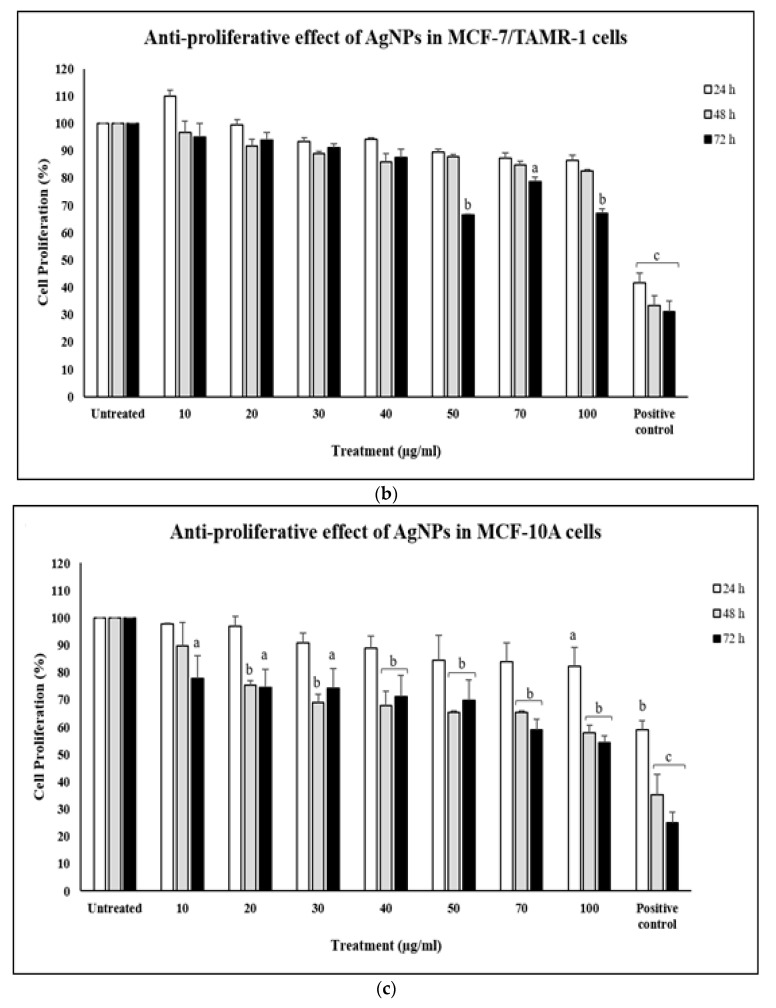
The anti-proliferative effect of commercial AgNPs on (**a**) MCF-7, (**b**) MCF-7/TAMR-1 and (**c**) MCF-10A cells within 24–72 h of treatment. The anti-proliferative effect of commercial AgNPs (10–100 µg/mL) was assessed by MTT assay. Tamoxifen (30 µM) was used as positive control. Data shown are the mean values ± S.D. for three independent experiments. Statistical analysis was performed using Student’s *t* test with ^a^ *p* < 0.05, ^b^ *p* < 0.01 and ^c^ *p* < 0.001, significantly different to untreated cells.

A commercially available AgNPs nanopowder was used as standard control for AgNPs-GA, and its anti-proliferative effect against MCF-7, MCF-7/TAMR-1 and MCF-10A cell lines are shown in Figure 9a–c. In comparison to biologically synthesized AgNPs from *G. atroviridis* leaf extract, commercial AgNPs inhibited 50% of MCF-7 cells only at concentrations higher than 70 µg/mL after 72 h of treatment (Figure 9a). In MCF-7/TAMR-1 cells, commercial AgNPs displayed about 30% growth inhibition only at concentration of 50 µg/mL after 72 h of treatment (Figure 9b). Only at the highest concentration tested in this study (100 µg/mL) did they display about 40% growth inhibition against normal cell line, MCF-10A cells after 72 h of treatment (Figure 9c). It was thus observed that commercial AgNPs were less cytotoxic in comparison to the biosynthesized AgNPs-GA. This could be due to a synergistic effect of the biosynthesized AgNPs-GA and their biological coating that may increase the inhibitory effect against MCF-7 and MCF-7/TAMR-1 cells.

According to the FTIR analysis findings it can be postulated that flavonoids and phenolic compounds present in *G. atroviridis* leaf extract, at least in part, may contribute to the aforementioned anti-proliferative effect of AgNPs-GA to both MCF-7/TAMR-1 and MCF-7 cell lines. Besides FTIR analysis, we had previously characterized the phytochemical contents of *G. atroviridis* leaf extract by using gas chromatography-mass spectrometry (GC-MS). (*E*)-β-Farnesene (58.5%) and β-caryophyllene (16.9%) were the most abundant sesquiterpene metabolites found in its leaves [17]. β-caryophyllene was previously demonstrated to potentiate the anticancer effects of paclitaxel on MCF-7, DLD-1 and L-929 cell lines [63].

**Figure 10:**
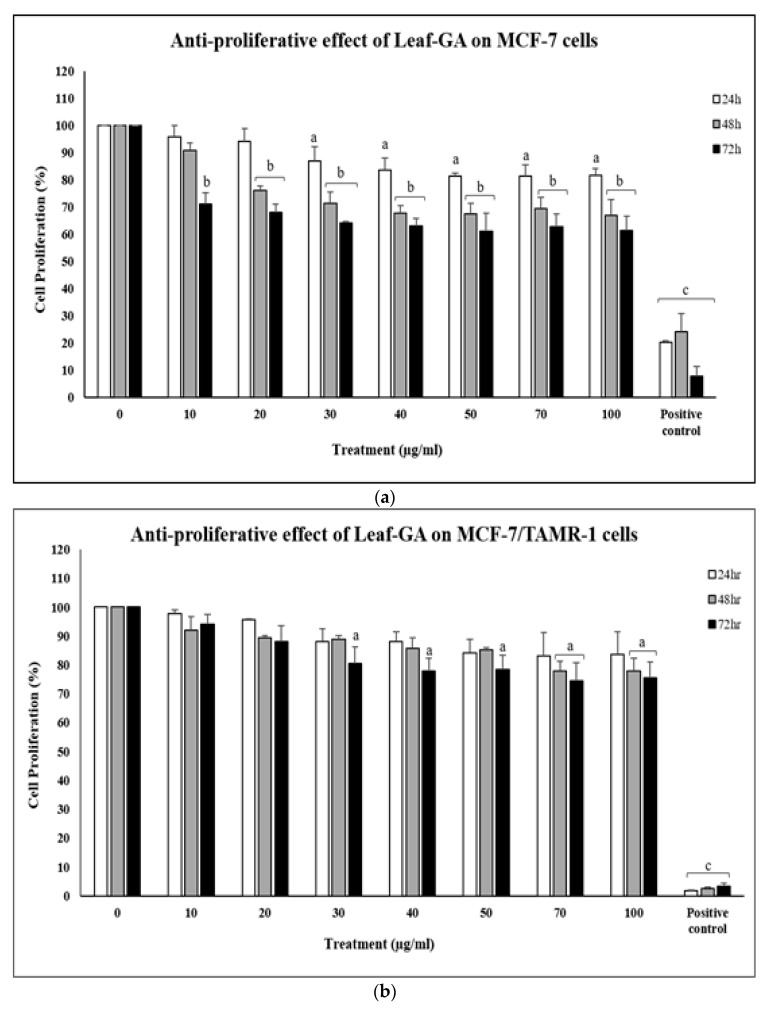

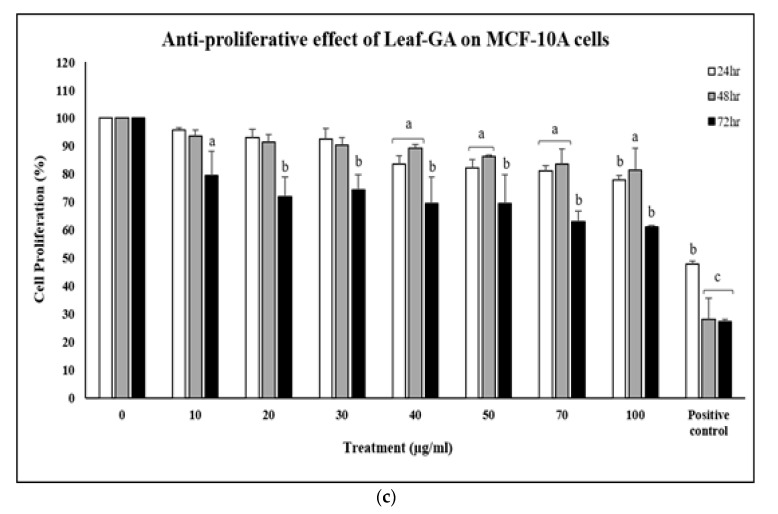
The anti-proliferative effect of Leaf-GA on (**a**) MCF-7, (**b**) MCF-7/TAMR-1 and (**c**) MCF-10A cells within 24–72 h of treatment. The anti-proliferative effect of commercial Leaf-GA (10–100 µg/mL) was assessed by MTT assay. Tamoxifen (30 µM) was used as positive control. Data shown are the mean values ± S.D. for three independent experiments. Statistical analysis was performed using Student’s *t* test with ^a^ *p* < 0.05, ^b^ *p* < 0.01 and ^c^ *p* < 0.001, significantly different to untreated cells.

Altogether, these findings provide additional evidence of the anti-proliferative effect of AgNPs-GA, through its synergistic action with the other plant metabolites presence in the leaf extract. Figure 10 shows the anti-proliferative effects of *G. atroviridis* leaf extract (Leaf-GA) against MCF-7, MCF-7/TAMR-1 and MCF-10A cell lines. In MCF-7 cells, a uniform of about 30% growth inhibition were observed within 10–100 µg/mL concentrations of Leaf-GA after 72 h of treatment (Figure 10a). In MCF-7/TAMR-1 cells, about 20% of growth inhibition were observed after 72 h of treatment with Leaf-GA at concentrations above 30 µg/mL (Figure 10b). In MCF-10A cells, 100 µg/mL of Leaf-GA displayed about 40% growth inhibition after 72 h of treatment (Figure 10c). Taken together, AgNPs-GA was found to be more cytotoxic than its leaf extract, Leaf-GA. The increased cytotoxicity of AgNPs-GA which was differed to corresponding bulk materials (Leaf-GA) even though they shared the same chemical composition. This can be due to nanostructuring of materials (AgNPs-GA) resulting in an amplified ratio of reactive surface atoms to inert core atoms and subsequent increased surface reactivity [55].

### 2.3. IC_50_ and SI Values of AgNPs-GA, Leaf-GA, Commercial AgNPs and Tamoxifen

Fifty percent of cell death values, which determine the inhibitory concentration (IC_50_) value of each treatment are summarized in Table 2 (left panel). The degree of selectivity of a cytotoxic agent towards targeted cancer cells in comparison to their normal counterparts can be expressed by its selectivity index (SI) value. SI values of each treatment were further calculated based on the IC_50_ value obtained in normal cells divided by IC_50_ value obtained in cancer cells [64,65]. SI values of AgNPs-GA, Leaf-GA, commercial AgNPs and tamoxifen are shown in Table 2 (right panel). Findings of the present study showed that AgNPs-GA was selectively cytotoxic to MCF-7 cells after 72 h of treatment, with SI value of 2.5. Even though AgNPs-GA possesses strong anti-proliferative effect against MCF-7/TAMR-1 cells, the SI value calculated for this cytotoxic agent was below than 2.0, which indicates its properties as a general toxin [66,67]. The SI values of tamoxifen in MCF-7 and MCF-7/TAMR-1 cells were 1.7 and 1.3, respectively, after 72 h of treatment. However, SI value for Leaf-GA and commercial AgNPs could not be calculated since neither treatment inhibited 50% of cell proliferation with the concentrations tested in this study. Studies have shown that cytotoxic agents with SI values of 2 and above are more toxic towards cancer cells in comparison to normal cells [66,67]. Based on the calculated IC_50_ values, these results suggest that AgNPs-GA is cytotoxic against both MCF-7 and MCF-7/TAMR-1 cells, when compared to Leaf-GA and commercial AgNPs. Based on the calculated SI values, AgNPs-GA generates general toxicity with no selectivity between breast cancer and normal cells, a potential vulnerability that could be improvised in future studies.

### 2.4. Comparison of Induction of Apoptosis in MCF-7, MCF-7/TAMR-1 and MCF-10A Cells Treated with AgNPs-GA, Leaf-GA and Commercial AgNPs with Annexin V-FITC and Propidium Iodide

**Figure 11:**
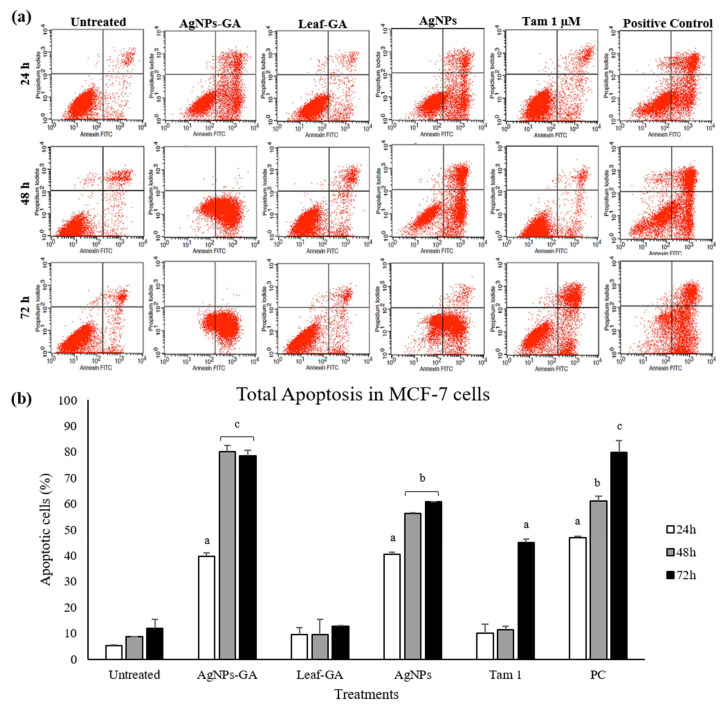
(**a**) Representative dot plot showing annexin-V-FITC^-^/PI^-^ (lower left quadrant/viable cells), annexin-V-FITC^+^/PI^−^ (lower right quadrant/early apoptotic cells), annexin-V-FITC^+^/PI^+^ (upper right quadrant/late apoptotic cells) and annexin-V-FITC^-^/PI^+^ (upper left quadrant/necrotic cells) in MCF-7 cells as acquired by flow cytometry. (**b**) Percentage of total apoptosis (early and late apoptosis) in MCF-7 cells after 24–72 h incubation period with medium alone (untreated), AgNPs-GA (15 µg/mL), leaf extract (100 µg/mL), AgNPs (100 µg/mL), tamoxifen (1 µM) and positive control (tamoxifen 30 µM). Data shown are the mean values ± S.D. for three independent experiments. Statistical analysis was performed using Student’s *t* test with ^a^ *p* < 0.05, ^b^ *p* < 0.01 and ^c^ *p* < 0.001, significantly different to untreated cells.

**Figure 12:**
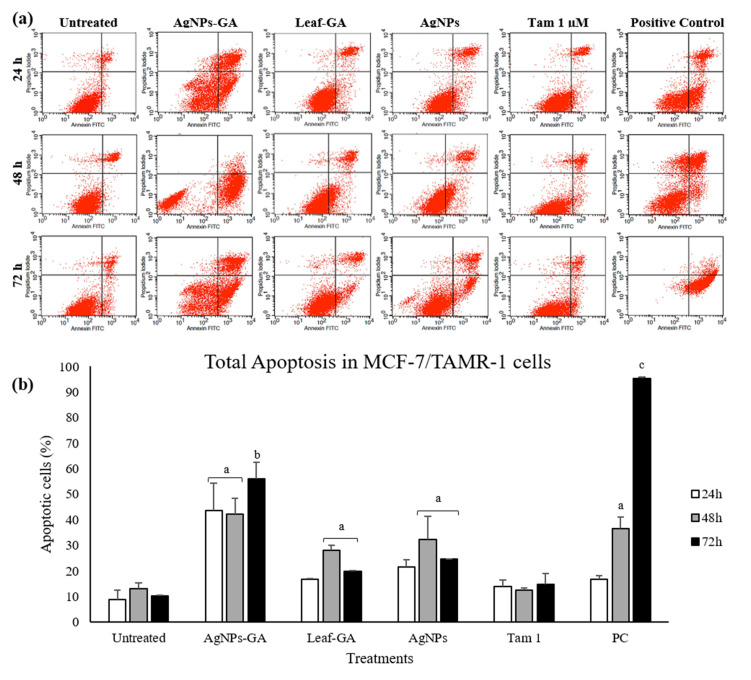
(**a**) Representative dot plot showing annexin-V-FITC^−^/PI^−^ (lower left quadrant/viable cells), annexin-V-FITC^+^/PI^−^ (lower right quadrant/early apoptotic cells), annexin-V-FITC^+^/PI^+^ (upper right quadrant/late apoptotic cells) and annexin-V-FITC^−^/PI^+^ (upper left quadrant/necrotic cells) in MCF-7/TAMR-1 cells as acquired by flow cytometry. (**b**) Percentage of total apoptosis (early and late apoptosis) in MCF-7/TAMR-1 cells after 24–72 h incubation period with medium alone (untreated), AgNPs-GA (15 µg/mL), leaf extract (100 µg/mL), AgNPs (100 µg/mL), tamoxifen (1 µM) and positive control (tamoxifen 30 µM). Data shown are the mean values ± S.D. for three independent experiments. Statistical analysis was performed using Student’s *t* test with ^a^ *p* < 0.05, ^b^ *p* < 0.01 and ^c^ *p* < 0.001, significantly different to untreated cells.

**Figure 13:**
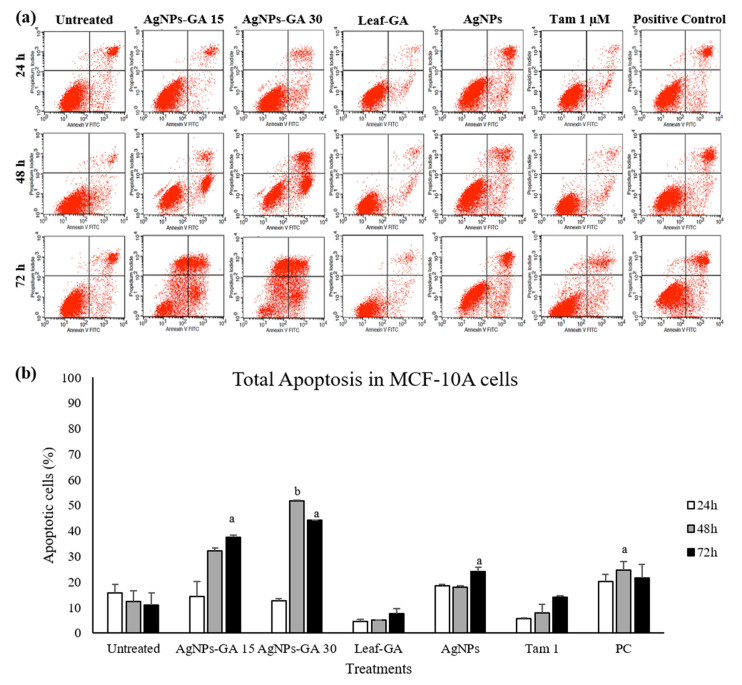
(**a**) Representative dot plot showing annexin-V-FITC^−^/PI^−^ (lower left quadrant/viable cells), annexin-V-FITC^+^/PI^−^ (lower right quadrant/early apoptotic cells), annexin-V-FITC^+^/PI^+^ (upper right quadrant/late apoptotic cells) and annexin-V-FITC^−^/PI^+^ (upper left quadrant/necrotic cells) in MCF-10A cells as acquired by flow cytometry. (**b**) Percentage of total apoptosis (early and late apoptosis) in MCF-10A cells after 24–72 h incubation period with medium alone (untreated), AgNPs-GA (15 and 30 µg/mL), leaf extract (100 µg/mL), AgNPs (100 µg/mL), tamoxifen (1 µM) and positive control (tamoxifen 30 µM). Data shown are the mean values ± S.D. for three independent experiments. Statistical analysis was performed using Student’s *t* test with ^a^ *p* < 0.05, ^b^ *p* < 0.01 and ^c^ *p* < 0.001, significantly different to untreated cells.

The induction of apoptosis, after the treatment with IC_50_ concentration of AgNPs-GA, Leaf-GA (100 µg/mL), commercial AgNPs (100 µg/mL) and tamoxifen (1 and 30 µM) were assessed by flow cytometry after staining with Annexin V-FITC and propidium iodide (PI). The flow cytometry results are shown in Figure 11, Figure 12 and Figure 13.

In Figure 11a, Figure 12a, and Figure 13a, the total percentage of cells located in lower and upper right quadrant regions were considered as the percentage of total apoptosis. These results were summarized in bar graphs as shown in Figure 11b, Figure 12b, and Figure 13b. At 24 h of treatment, the rate of total apoptosis (early and late apoptosis) in MCF-7 cells treated with AgNPs-GA, Leaf-GA and commercial AgNPs were 39.77 ± 1.3%, 9.55 ± 2.6% and 40.52 ± 0.9%, respectively (Figure 11b). The percentage of apoptotic cells was significantly increased to 80.15 ± 2.3% and 78.35 ± 2.2% in MCF-7 cells treated with AgNPs-GA after 48 h and 72 h of treatment, respectively.

Commercial AgNPs resulted in 56.16 ± 0.3% and 60.91 ± 0.01% of apoptosis in MCF-7 cells after 48 h and 72 h of treatment, respectively. On the contrary, there was no significant increase in the percentage of apoptotic cells by Leaf-GA compared to the untreated cells. In MCF-7/TAMR-1 cells, total apoptosis observed was 43.69 ± 10.5%, 16.71 ± 0.3% and 21.46 ± 3% after 24 h treated with AgNPs-GA, Leaf-GA and commercial AgNPs, respectively (Figure 12b).

A higher rate of apoptosis was recorded after 72 h treatment with AgNPs-GA than Leaf-GA and commercial AgNPs in this cell line. At 72 h of treatment, AgNPs-GA caused 56.14 ± 6.4% of apoptosis, whilst Leaf-GA and commercial AgNPs demonstrated 19.8 ± 0.3% and 24.57 ± 0.2% of apoptosis induction in MCF-7/TAMR-1 cells. These results clearly indicated that AgNPs-GA potently inhibited the growth of MCF-7/TAMR-1 cells through the mechanism of apoptosis. In MCF-10A non-cancerous human breast cell line, total apoptosis induced by AgNPs-GA, Leaf-GA and commercial AgNPs was 37.6 ± 0.7%, 7.7 ± 1.8% and 24.12 ± 1.7%, respectively, after 72 h of treatment (Figure 13b).

At similar incubation period, the percentage of apoptotic cells was 44.15 ± 0.1% in MCF-10A cells with AgNPs-GA at twice IC_50_ concentration (30 µg/mL). Previous studies have demonstrated that the physicochemical and structural properties of AgNPs play a major role in their interactions with cells [53]. Furthermore, variations in these properties among different AgNPs may result in different toxicological effects.

The mechanism responsible for AgNPs’ toxicity to human cells have been reported to involve with decrease membrane integrity in a variety of human cell lines such as HeLa [68]. During apoptosis, cells undergo characteristic morphological destructions, like the loss of membrane integrity. This in turn, causes the translocation of phosphatidylserine (PS) to the outer leaflet of the plasma membrane. In the present study, the appearance of PS in apoptotic cells was detected by Annexin V-FITC. Taken together, our findings may provide ample evidence that AgNPs-GA possessed its anti-proliferative effect at least in part, associated with the activation of apoptosis.

## 3. Materials and Methods

### 3.1. Plant Material and Leaf Extract Preparation

Leaves of *G. atroviridis* were collected from Kepala Batas (Penang, Malaysia). A voucher specimen (11764) has been deposited with the herbarium of Universiti Sains Malaysia, Penang, Malaysia. Fresh leaves were washed with distilled water and the debris removed. Then, the leaves were dried in an industrial hot air oven at 40 °C for 3 days and ground using an herb grinder. Briefly, 10 g leaf powder was mixed with 100 mL deionised water in a beaker. The mixture was warmed in a water bath at 60 °C for 10 min. After cooled down at room temperature, the crude extract was filtered using Whatman filter paper No. 1 and the filtrate was stored at 4 °C for further use.

### 3.2. Biosynthesis of Silver Nanoparticles (AgNPs-GA)

In the present study, AgNPs-GA were biosynthesised using a previously reported method with a slight modification. Several parameters which include concentration of silver salt (AgNO_3_) and *G. atroviridis* leaf extract, mixing ratio of reactants, temperature and pH were optimised in the course of AgNPs-GA. The concentration range of AgNO_3_ (Nacalai Tesque Chemicals, Nakagyo-ku Kyoto, Japan) and leaf extract used in this study were 0.001–0.1 M and 5–20% (*w*/*v*), respectively. The mixing ratio of AgNO_3_ to leaf extract was optimised at 1:4, 1:9 and 2:3 with 100 mL as final volume. The optimal reaction time was determined from 24 h to 72 h of incubation period. The concentration of AgNO_3_, leaf extract and mixing ratio of reactants that yield an absorption peak at 400–450 nm in the UV-Visible spectrum were further assessed across different temperatures (room temperature, 32 °C, and 37 °C). The pH of reaction mixture were also measured in order to optimise the yield and properties (e.g., size and shape), of AgNPs-GA, Similarly, the change in color of the reaction mixture from light yellow to dark brown was also observed and the samples were subjected to characterisation.

### 3.3. Characterization of Silver Nanoparticles

Silver nanoparticles (AgNPs-GA) were purified by centrifugation at 10,000 rpm for 15 min and the pellets were thoroughly washed (thrice) with deionised water and resulting colloidal suspension was characterised using various analytical instruments as described below.

#### 3.3.1. Ultra Violet Visible (UV-Vis) Spectroscopy

UV-Vis spectra was measured using a Lambda 25 UV-Vis spectrophotometer (Perkin Elmer, Waltham, MA, USA). All measurements were performed within the range of 350–600 nm with 1 nm intervals.

#### 3.3.2. Scanning Electron Microscopy (SEM)

The morphology of AgNPs was studied using scanning electron microscopy (FEI SEM, Quanta FEI 650, Field Electron and Ion Company, Hillsboro, OR, USA) at an accelerated voltage of 15 kV. One drop of the dispersion containing AgNPs was placed on a small aluminium plate and dried at room temperature. The dried AgNPs were then coated with gold metal under high vacuum and examined. Representative SEM images were reported.

#### 3.3.3. Transmission Electron Microscopy (TEM)

Bright field TEM images for AgNPs were obtained using a transmission electron microscope (Philips CM12, FEI Ltd., Eindhoven, The Netherlands) operated at 120 Kv. Images were recorded using a SIS MegaView III digital camera (SIS Analytics, Hamburg, Germany) and analyzed with software ImageJ. To prepare samples for TEM, AgNPs was dissolved in 90% ethanol and sonicated for 3 min. Then, a drop of AgNPs suspension was placed on a carbon-coated copper grid. The sample was allowed to air-dry for 3 min at room temperature and the grid is examined with TEM.

#### 3.3.4. The Surface Zeta Potential Distribution of Silver Nanoparticles/Dynamic Light Scattering Analysis (DLS)

Size distribution and zeta potential of bio-reduced AgNPs were measured using DLS (Zetasizer Nano ZS, ZEN3600, Malvern Instruments Ltd., Malvern, Worcestershire, UK). The mean size and its zeta potential of the particles were obtained.

#### 3.3.5. X-ray Diffraction (XRD)

X-ray diffraction measurement was carried out to determine the crystallographic structure and crystallite size (grain size) of AgNPs. The freeze dried AgNPs were coated onto XRD grid and analyzed using an X-ray diffractometer (X’Pert-Pro, PANalytical, Veeco, New York, NY, USA) operated at 40 kV and a current of 30 mA with Cu Kα radiation at 2θ angel. The scanning was performed in the region of 2θ from 20° to 80°.

#### 3.3.6. Fourier-Transform Infrared (FTIR) Spectroscopy

The FTIR analyses were recorded on a Perkin Elmer FTIR spectrophotometer (Perkin Elmer, Waltham, MA, USA). Spectra were collected at a spatial resolution of 4 cm^−1^ in the transmission mode, between 4000 and 400 cm^−1^, respectively.

### 3.4. Cell Culture

The human breast cancer cell lines MCF-7 and MCF-7/TAMR-1 were purchased from the American Type Culture Collection (ATCC, Rockville, MD, USA) and Merck (Darmstadt, Germany), respectively. MCF-7 cells were routinely cultured in Roswell Park Memorial Institute (RPMI) 1640 medium (Gibco, Burlington, ON, Canada) supplemented with 10% fetal bovine serum (FBS, Gibco) while MCF-7/TAMR-1 cells were routinely cultured in phenol red-free DMEM/F12 medium (Gibco) supplemented with 1% FBS, 6 ng/mL insulin (Gibco) and 1 µM tamoxifen (Nacalai Tesque) following the manufacturer’s recommendation. The cell cultures were maintained at 37 °C in a humidified atmosphere with 5% CO_2_ to reach 70–80% confluence. The monolayer cells were detached with trypsin–ethylene diamine tetra acetic acid (trypsin-EDTA, Gibco) to prepare single cell suspensions.

#### 3.4.1. Cell Proliferation Assay

The anti-proliferative effect of biosynthesised AgNPs-GA in MCF-7 and MCF-7/TamR-1 cancer cells was analysed using 3-(4, 5-dimethylthiazol-2-yl)-2,5-diphenyltetrazolium bromide (MTT) assay (Sigma Aldrich, St. Louis MO, USA). The MTT assay measures cell metabolic activity based on the reduction of tetrazolium dye MTT into its insoluble purple-colored formazan. The amount of formazan is directly proportional to the number of viable cells and inversely proportional to the cytotoxicity degree. MCF-7 cells were seeded at a concentration of 2 × 10^4^ cells/100 µL complete medium in 96-well flat-bottomed microtiter plates and left to adhere for 24 h at 37 °C in 5% CO_2_ incubator. MCF-7/TAMR-1 cells were seeded at a concentration of 5 × 10^4^ cells/100 µL complete medium and left to adhere for 48 h at 37 °C, 5% CO_2_. The complete medium was changed and cells were treated with AgNPs-GA (10–100 μg/mL). Tamoxifen (30 µM) and culture medium were also included which served as positive and negative controls, respectively. Three replicates were performed for each treatment and controls. The plates were further incubated for 24, 48 and 72 h at 37 °C, 5% CO_2_. At each incubation period, MTT solution with volume of 10 μL (5 mg/mL) was added into each well and incubated at 37 °C, 5% CO_2_ for another 4 h. The solution in each well containing media, unbound MTT and dead cells were aspirated and 100 µL dimethyl sulphoxide (DMSO, QReC^TM^, Auckland, New Zealand) was added to each well to solubilize the purple formazan crystals. The plate was placed on shaker for 2 min to thoroughly dissolve the MTT color product. The reduction of the MTT solution was determined spectrophotometrically at 570 nm with 630 nm as reference wavelength using a microplate reader (PowerWaveXS, Bio-Tek, Winooski, VT, USA). The optical density (O.D.) value of the solution directly represents relative cell numbers. The O.D. values were converted into percentages of cell proliferation using the following formula:

Cell proliferation (%) = [(O.D. treatment − O.D. blank)/(O.D. untreated cell − O.D. blank)] × 100%(1)

#### 3.4.2. Selectivity Index (SI)

The degree of selectivity of the cytotoxic agent was expressed by its SI vaue as suggested by Badisa et al. [64]. A high SI value (>2) of an agent suggests selective toxicity against cancer cells, while an agent with SI value < 2 is considered to give general toxicity which can cause cytotoxicity in normal cells [65]. Each SI value was calculated using the formula:

SI = IC_50_ normal cell/IC_50_ cancer cell (2)

### 3.5. Apoptosis Assay

Annexin V-FITC was used as a marker of phosphatidylserine exposure and propidium iodide (PI) as a marker for dead cells (FITC Annexin V Apoptosis Detection Kit I; BD BiosciencesSan Jose, CA, USA). This combination allows differentiation among viable cells (annexin V-negative, PI-negative), early apoptotic cells (annexin V-positive, PI-negative), late apoptotic cells (annexin V-positive, PI-positive) and necrotic cells (annexin V-negative, PI-positive). Each cell line was seeded and treated with AgNPs-GA (15 µg/mL) or Leaf-GA (100 µg/mL) or commercial AgNPs (Sigma Aldrich, 100 µg/mL) or tamoxifen (1 and 30 µM) or culture medium alone as negative control (untreated cells). The cells were incubated for 24–72 h of treatment prior flow cytometric analysis. At each incubation period, cells were harvested by centrifugation and washed twice with phosphate buffer saline (PBS). Aliquots of 1 × 10^6^ cells/mL were resuspended in 1 × binding buffer provided with the kit. Hundred microliters of the solution (1 × 10^5^ cells) were transferred into a flow tube. A volume of 5 µL of Annexin V-FITC and 5 µL of PI were added and cells were incubated at room temperature in the dark for 15 min. Following this, 400 µL of 1 × binding buffer were added into each tube prior flow cytometry acquisition and analysis. A minimum of 20,000 cells were analyzed by using FACS Calibur flow cytometry and CellQuest Pro software (Becton Dickinson, Franklin Lakes, NJ, USA).

### 3.6. Statistical Analysis

Data were expressed as mean ± standard deviation (S.D.) and all the experiments were repeated independently three times. Statistical differences between control versus treatment for all experiments were determined using Student’s t–test.

## 4. Conclusions

The important findings of this study include the biological technique used to synthesise AgNPs using *G. atroviridis* leaf extract followed by the characterization of AgNPs-GA formation. The leaf extract from *G. atroviridis* was found to be highly capable of producing AgNPs with favourable physicochemical and biological properties. In this study, AgNPs-GA were biosynthesized from the silver salt through the reducing power of phenolic compounds present in the leaf extract. This may suggest that *G. atroviridis* leaf extract works synergistically with its bioactive molecules and could be used as an efficient natural bioreducing agent for the production of silver nanoparticles. The various parameters including silver salt and leaf extract concentrations, mixing ratio, pH, temperature and reaction time exert an important role in the formation of AgNPs-GA. Various analytical techniques were used to characterize the newly biosynthesized AgNPs-GA and their size was determined to be 5–30 nm. The findings of the present study also showed that AgNPs-GA were spherical and face-centered-cubic in shape. The method of AgNPs-GA synthesis introduced in this study, therefore, holds great potential as a simple, low-cost, and environmentally friendly approach. This report also emphasizes that AgNPs-GA exert selective anti-proliferative and apoptotic effects towards human breast cancer MCF-7 and MCF-7/TAMR-1 cells when compared to their effect on the normal cell line tested. Furthermore, the biosynthesised AgNPs-GA were proved to possess improved anti-proliferative activity by inducing apoptosis in MCF-7 and MCF-7/TAMR-1 breast cancer cells in comparison with commercial AgNPs and leaf extract. Taken together, these findings imply that the biological synthesized AgNPs using leaf extract holds promising approach and potent candidate against breast cancer, particularly to those who are tamoxifen-resistant, however details mechanism of its actions would be needed.

## Figures and Tables

**Table 1:** Summary of various optimized reactions condition for the biosynthesis AgNPs-GA.

Optimized Reactions Condition	Optimum Values
Concentration of AgNO_3_	0.1 M
Concentration of *G. atroviridis* leaf extract (Leaf-GA)	10% (*w/v*)
Mixing ratio of reactants	1:4 (ratio of Leaf-GA to AgNO_3_)
Incubation temperature of the medium	32 °C
pH of the medium	3
Incubation time	72 h

**Table 2:** IC_50_ and SI values of AgNPs-GA, Leaf-GA, commercial AgNPs and tamoxifen in MCF-7, MCF-7/TAMR-1 and MCF-10A cell lines.

Treatment/Time Point	Cell Line/Average of IC_50_ (µg/mL)			Selective Index (IC_50_ in Normal Cells/IC_50_ in Cancer	
	**MCF-7**	**MCF-7/TAMR-1**	**MCF-10A**	**MCF-7**	**MCF-7/TAMR-1**
**AgNPs-GA**					
24 h	15.0	34.0	7.0	0.5	0.2
48 h	8.0	34.0	6.0	0.8	0.2
72 h	2.0	32.0	5.0	2.5	0.2
**Leaf-GA**					
24 h	>100	>100	>100	-	-
48 h	>100	>100	>100	-	-
72 h	>100	>100	>100	-	-
**AgNPs (commercial)**					
24 h	>100	>100	>100	-	-
48 h	100	>100	>100	-	-
72 h	58.0	>100	>100	-	-
**Tamoxifen**					
24 h	22.5	>30	17.0	0.8	-
48 h	14.0	22.0	17.0	1.2	0.8
72 h	8.8	11.5	15.0	1.7	1.3
